# Michigan State Board of Health

**Published:** 1877-11

**Authors:** 


### MICHIGAN STATE BOARD OF HEALTH.

The regular quarterly meeting of the Michigan State Board of Health was held in Lansing, on Tuesday, Oct. 9th. The members present were: Prof. R. C. Kedzie, Lansing; Homer O. Hitchcock, M. D., Kalamazoo; Henry F. Lyster, M. D., Detroit; Hon. LeRoy Parker, Flint; Rev. D. C. Jacokes, Pontiac; Henry B. Baker, Secretary.

Dr. Kedzie, committee on poisons, etc., read an important report on "Labeling Medicines." He gave many instances of poisoning by taking the wrong medicine through mistake because it was not labeled. He urged that every medicine, and every injurious substance which may be mistaken for medicine, should be distinctly labeled. "Never administer as medicine any substance of the composition of which you are ignorant or in doubt." The paper was accepted with thanks, and a committee, of which Dr. Kedzie is chairman, was appointed to investigate another branch of the subject in reference to danger from the dispensing of drugs or medicines by unqualified or inexperienced persons. This committee is to confer with the Michigan Pharmaceutical Association, which has already given attention to the subject.

Dr. Baker presented tables, diagrams, etc., on the subject of the death rate as relates to age, climate, etc. He gave tables and diagrams for the various States and Territories of the Union.

Leroy Parker, chairman of the committee on legislation, made a brief report relative to the subject of boards of health in cities and villages, and mentioned that since the last meeting considerable progress had been made in securing health officers for such boards. The secretary stated that the progress in this direction had been great, and it was largely due to Mr. Parker's efforts.

Dr. H. F. Lyster read a continuation of his paper heretofore presented on the subject of "Healthy Homes." He considered the subject mainly with reference to their location and the measures to be taken to secure good drainage, and traced much of the ill-health of people to dampness in and about their dwellings. He had issued a circular to the correspondents of the board, and with this paper he presented the substance of about forty replies received, showing the nature of the soil, practice as to tile-draining, sources of drinking water, character of cellars, disposition of decomposing organic matter, etc., about the homes in the several localities. He recommended that wherever the soil is not dry, there should be tile drains around the house or under the cellar.

In the discussion which followed, Dr. Baker deemed it important that such drains should never communicate uninterruptedly with a sewer, which may contain sewer gas, which will thus permeate the house; but the connection should be through an open-air space, or otherwise freely ventilated on the house side of the trap.

Dr. Kedzie said that if box drains be used, they should be placed with one corner down, so as to be self-cleansing.

Dr. Kedzie read a paper on persistence in efforts to resuscitate the drowned. He reported a large number of cases where persons had been resuscitated a long time after they had apparently ceased to live. He claimed that deaths are constantly occurring for lack of thorough efforts at resuscitation, and that whenever such efforts are made they should be continued at least two hours. He cited one instance where only after six hours of constant work did symptoms of life appear, and yet this person was completely restored.

The Secretary read an outline of a report of the work of his office during the last quarter. It included the distribution of about five thousand copies of the document on "Restriction and Prevention of Scarlet Fever," sixteen hundred copies of the Fourth Annual Report of the Board; and the printing of six thousand copies on the "Treatment of the Drowned." Much time had been given to the compilation of "Weekly Reports of Diseases," and a large amount of miscellaneous correspondence and other business had been transacted.

Hon. Leroy Parker read an abstract of papers read before the public health section of the American Social Science Association at Saratoga, which he had attended in the interests of public health in Michigan.

Dr. Hitchcock presented a report and abstract of papers read at the recent meeting of the American Public Health Association at Chicago.

At the last meeting ex-President Hitchcock presented an address by title, and at this meeting it was read. The subject was: "Heredity in its relations to the public health, and to legislation in the interests of public health."

A valuable paper on the diet of infants, by Dr. Arthur Hazlewood, of Grand Rapids, an ex-member of the Board, was accepted with thanks.

The Secretary read communications from Dr. G. W. Topping, of DeWitt, relative to reports of prevailing diseases; from Dr. O. Marshall, of this city, on the subject of opium-eating; from Dr. Edward Dorsch, of Monroe, on lead-poisoning from tin cooking utensils lined or glazed with lead; from Dr. C. W. Marvin, of Ithaca, relative to the recent increase of deaths from cancer; from Dr. J. D. Hull, of Allegan county, relative to drainage in his locality; from Dr. Batwell, of Ypsilanti, relative to sickness from damming the Huron river; from Dr. Charles H. Fisher, of Rhode Island, giving formula for preparation and an account of the first use of sulpho-carbolate of soda as a preventive in scarlet fever.

Dr. Lyster presented a paper on baths and bathing. He gave a history and description of all kinds of baths and their effects on the human body. His paper was also accompanied by numerous replies on this subject from correspondents of the Board to a circular which he had issued.

The Secretary presented his annual report in relation to the property of the Board.

During the year additions had been made to the library, 97 by purchase and 244 by gift and exchange. The library has now 931 numbers.

